# Mycotoxin Incidence in Some Fish Products: QuEChERS Methodology and Liquid Chromatography Linear Ion Trap Tandem Mass Spectrometry Approach

**DOI:** 10.3390/molecules24030527

**Published:** 2019-02-01

**Authors:** Josefa Tolosa, Francisco J. Barba, Guillermina Font, Emilia Ferrer

**Affiliations:** Laboratory of Food Chemistry and Toxicology, Faculty of Pharmacy, University of Valencia, Avenue Vicent Andrés Estellés s/n, 46100 Burjassot, Spain

**Keywords:** smoked salmon, sushi, fish, mycotoxins, liquid chromatography, mass spectrometry

## Abstract

The inclusion of vegetal raw materials in feed for fish farming has increased the risk of mycotoxin occurrence in feed, as well as in edible tissues from fish fed with contaminated feed, due to the carry-over to muscle portions. Therefore, the objective of this study was to evaluate the occurrence of 15 mycotoxins in processed fish products, which are commonly consumed, such as smoked salmon and trout, different types of sushi, and gula substitutes. A QuEChERS method was employed to perform the mycotoxin extraction from fish samples. For mycotoxin identification and quantitation, the selected technique was the liquid chromatography-tandem mass spectrometry linear ion trap (LC-MS/MS-LIT). Smoked fish and sushi samples results were negative regarding the presence of all 15 mycotoxins studied. In contrast, small amounts of fusarenon-X and enniatin B were found in gula substitute samples.

## 1. Introduction

Over the last years, there has been an ongoing interest in products from aquaculture farming, due to the increasing demand for aquatic products and seafood. The specific reasons for this increase are, on the one hand, the nutritional benefits of fish consumption regarding polyunsaturated fatty acids, such as omega-3, and on the other hand, the increasing demand for ready to eat food products worldwide, thus requiring less preparation before serving, which supposedly has an important change in the preservation and processing of different fish-derived products [1]. In addition, some products have been exported to different countries, due to the globalization process, as occurs with sushi and sashimi, which are originally from Japan. Thus, a wide variety of different fish products produced by diverse cooking and preservation techniques, such as surimi, gula substitutes, and several types of sushi are available in the global market [2].

However, the ingredients included in the elaboration of these products (mainly from vegetal sources) or the carry-over of contaminants from feed to edible tissues of farmed fish can constitute a source for mycotoxin contamination in these products. For instance, common ingredients used in gula substitute elaboration, mainly fish protein, wheat flour, soya, and vegetal protein, can introduce mycotoxins previously present in those raw materials in these kind of foodstuffs during the elaboration process. The same can occur with sushi. Sushi is a Japanese dish of rice prepared with vinegar and some sugar and salt, commonly accompanied with rawor smoked fish or vegetables (such as different algae types) [2]. Regarding smoked fish products, there are diverse methods to preserve them. Some traditional techniques are mainly based on the control of temperature. However, the control of water activity (a_w_) is the key processing parameter for other techniques, such assalting, smoking, drying, and freeze-drying. Some of these techniques have been used since ancient times (e.g., smoked salmon) and are still the basis for products which are highly consumed nowadays [3]. For instance, an increase in smoked salmon consumption has been noticed in recent years, due to aquaculture development of the Atlantic salmon (*Salmo salar*).

On the other hand, the use of smoking, combined or not with salting, is also used as a preservation technique. This effect is attributed to the different compounds found in smoke from wood burning, which mainly consist of a large number of phenols, having anti-bacterial and anti-fungal properties, and being able to inhibit or destroy spoilage microorganisms, and thus, preserve the fish longer [4]. It combines three different effects: drying, cooking, and the effect of the smoke. However, the resulting smoked product, despite the protective effect of wood smoking process, is prone to fungi growth and contamination, mainly due to their low moisture, which can produce mycotoxins, especially during improper storage conditions [5].

Mycotoxins are toxic contaminants found in vegetal raw materials, mainly cereals and their by-products. In this sense, the use of contaminated cereals and vegetal ingredients in feed formulation can introduce these contaminants in food and feed chain [6]. For this reason, mycotoxins are frequently monitored in both food and feed within national and international public health programs. Among their occurrence in feed, animal derived products and edible tissues can be also contaminated with mycotoxins due to the animal ingestion of contaminated feed and their carry-over to those portions. Thus, animal by-products can constitute a potential source of mycotoxins [7,8,9,10]. Although some previous studies have evaluated the presence of mycotoxins in terrestrial animals, to the best of our knowledge, there is scarce information about mycotoxin determination on farmed fish compared to mycotoxin determination in livestock (mainly pigs and ruminants) [11]. Therefore, taking into account that some surveys have evaluated the mycotoxin occurrence on feed for farmed fish and in edible tissues of farmed fish, it is of great importance to evaluate mycotoxin occurrence in aquaculture products [6,12,13,14].

Currently, only a few mycotoxins frequently detected in agricultural commodities are regulated within the European Union. Thus, regarding foodstuffs, limit values have been set for some mycotoxins in Commission Regulation 1881/2007 [15] and its amendments, setting maximum residue limits for aflatoxins (AFs), ochratoxin A (OTA), patulin (PAT), deoxynivalenol (DON), zearalenone (ZEN), and fumonisins (FBs).

On the other hand, regarding feedstuffs, aflatoxin B1 (AFB1) is the only mycotoxin under European feed regulation until now (20 µg/kg in raw materials) [16], while for other mycotoxins, mainly *Fusarium* mycotoxins, guidance values have been set for feed ingredients and finished feed, including DON, ZEN, and the sum of fumonisin B1 and B2 (FB1 + FB2) [17], whereas for T-2 and HT-2 toxins, only indicative levels for cereal products have been set [18].

Within this context, the purpose of this work was to evaluate the mycotoxin occurrence in: (i) smoked salmon (*Salmo salar*), (ii) gula substitutes, and (iii) different types of sushi samples, which are commercially available. For this purpose, a QuEChERS extraction procedure was used for the simultaneous multi-mycotoxin evaluation of aflatoxins (AFB1, AFB2, AFG1, AFG2), fumonisins (FB1, FB2, FB3), enniatins (ENN A, ENN A1, ENN B, ENN B1), beauvericin (BEA), fusarenon-X (FUS-X), sterigmatocistin (STG), and ochratoxin A (OTA). The mycotoxin determination was carried out using liquid chromatography equipment coupled to tandem mass spectrometry with a linear ion trap (LC-MS/MS-LIT). To the best of our knowledge, this is the first study evaluating mycotoxin occurrence in this kind of foodstuffs.

## 2. Results and Discussion

### 2.1. LC-MS/MS-LIT Optimization and Method Validation

The method was optimized in terms of specificity, selectivity, linearity, sensitivity, trueness, and precision and matrix effect. Mycotoxin identification and quantitation was performed with a 3200 QTRAP^®^ System AB Sciex (Applied Biosystems, Foster City, CA, USA) functioning as a triple quadrupole mass spectrometer detector (MS/MS). Prior to optimizing the MS/MS conditions, full scan and daughter scan under positive mode were used. The MS was operated in MRM mode with the resolution set to unit resolution for Q1 and Q3. Thus, the mass spectrometric conditions were optimized by direct infusion of individual working standard solutions, using an ESI source in both positive and negative modes. Good sensitivity was obtained for selected mycotoxins when the ESI+ mode was applied, so analyses were carried out in ESI+ mode. For all analytes, entrance potential was set at 10V, while for collision cell exit potential (CXP), different values were selected. The cone voltage was optimized for each target mycotoxin and the most intense precursor ions were selected with the mass spectrometer operating in product ion scan mode. Subsequently, collision energies were optimized for each transition and product ions were selected for mycotoxin quantitation and qualification purposes. Table 1 shows the optimized parameters for each mycotoxin, as well as the 2 most relevant transitions in MRM mode.

The specificity and selectivity of the method relies on the chromatographic retention time (RT) of each analyte and on the SRM transition used. Thus, RT was used for the characterization of each compound, being the criteria selected for accepting the analysis of RT deviation < 2.5% compared to the standard in solvent [19].

Concerning linearity, all the studied mycotoxins showed correlation coefficients (R^2^) greater than 0.990 over the working range. To evaluate the linearity, calibration curves were prepared for each studied mycotoxin using either the standards prepared in MeOH and MeCN or those prepared in extracts of blank samples (smoked salmon, and gula substitute samples).

In order to carry out the recovery assays and to develop the calibration curves, smoked salmon and gula substitute samples were first analyzed to verify that no mycotoxin contamination was found, being these used as blank samples. Sushi samples analyzed in the study included smoked salmon and other ingredients also present in gula substitute samples (described in material and methods section). For this reason, smoked salmon and gula substitute samples were used as blank extracts. Then, these samples were spiked at two addition levels (10xLOQ and 100xLOQ) and they were kept at room temperature prior to extraction in order to allow solvent evaporation. Results of method validation are shown in Table 1 and Table 2.

The method sensitivity was assessed by determining the limit of detection (LOD) and the limit of quantitation (LOQ). LODs and LOQs were calculated using a blank sample extract fortified using decreasing analyte concentration and criteria signal to noise (S/N) ≥ 3 and S/N ≥ 10 for LOD and LOQ, respectively (Table 2).

The trueness, expressed in terms of recovery percentage, was evaluated at two spiking levels (low level: 10 × LOQ; high level: 100 × LOQ). Intra-day and inter-day precision were also evaluated by spiking the standard solution to samples at two spiked levels (Table 2). Regarding intraday precision, RSDr for the validated procedures at each spiked level were lower than 10 and 12%, respectively, while for interday precision, RSDr were lower than 13 and 15%, respectively. All the analyses were performed in triplicate and results expressed as the mean value for three replicates.

Co-eluting matrix components can negatively influence the accuracy of quantitative methods through signal ion suppression or enhancement (SSE) in the ion source, thus producing the so-called matrix effect (ME). For this reason, the effects of possible matrix mismatch were assessed in this survey. To minimize the ME, matrix-matched calibration curves were prepared for mycotoxin quantitation purposes in the fish derived samples included in the study. Then, the slopes of calibration curves prepared in solvent and in a matrix extract were compared between them to obtain the ME values, and results were expressed as signal suppression/enhancement (SSE). Matrix-matched calibration curves were made by using fortified samples with 7 addition levels, and then were used for mycotoxin quantitation. Regarding matrix effect studies, different percentages were observed (ranging from 56% to 95%, for FB1 and FUS-X, respectively). The higher recovery percentage (>100%) observed for some mycotoxins could be explained by the higher ME of these mycotoxins. Therefore, to minimize these matrix effects, especially in the case of FBs, matrix-matched calibration is required, especially for selective and reliable mycotoxin quantitation.

Taking into account the validation parameters obtained and optimized, it can be set so that performance characteristics fulfill the criteria set at Commission Decision 2002/657/EC [20] and guidance document on identification of mycotoxins in food and feed SANTE/12089/2016 [19]. Thus, based on the obtained validation results, the proposed procedure is suitable for its purpose, since it is a specific, sensitive, accurate, precise, and robust method.

### 2.2. Extraction Procedure

The use of QuEChERSfor extraction has been shown as a useful tool to be applied in different food matrices. For instance, QuEChERS has been widely employed to perform mycotoxin extraction in different food matrices [21]. In addition, compared to other extraction methods, QuEChERS has the advantage of being able to simultaneously extract the different targeted mycotoxins, making it adequate for multi-mycotoxin determination.

The selection of QuEChERS parameters was based on other previous studies [22,23], however, taking into account the complexity of the matrices studied in those studies, containing lipids, proteins, and carbohydrates, an additional step with C18 salt was necessary in order to obtain suitable recovery and minimal ME. For this reason, 0.1 g of C18 were added to the extract. Furthermore, a solution of 2% aqueous formic acid was necessary before MeCN addition to improve mycotoxin extraction [23].

### 2.3. Application to Food Matrices

**Figure 1:**
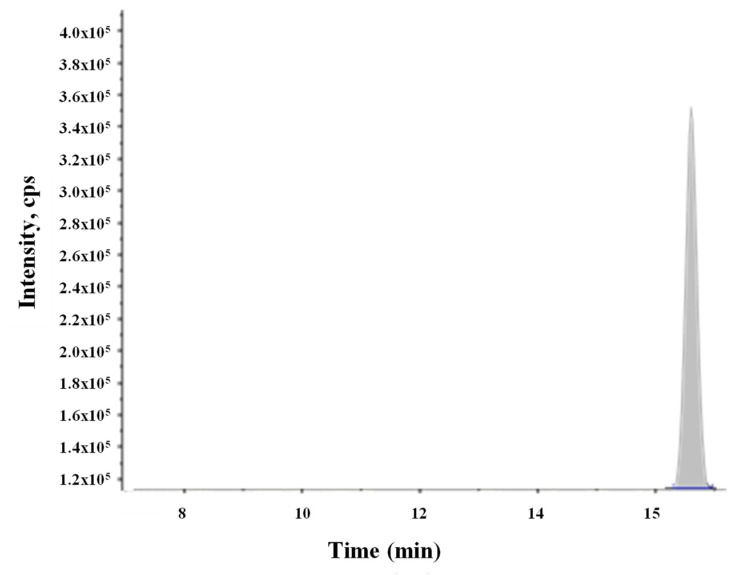
Showing ENN BB added to a gula substitute sample.

The above described extraction method was applied to 72 fish products to analyze mycotoxin contamination in that kind of foodstuffs. Mycotoxin determination was evaluated, and as in our previous study [14], the presence of ENNs in the salmon and trout samples, exclusively from aquaculture farming, was expected. In that study, ENNs were present in fish flesh of fish species—salmon and trout (20% of salmon and 10% of rainbow trout). However, in the present survey, smoked fish and sushi samples results were negative regarding the presence of all 15 mycotoxins studied, including ENNs. In contrast, gula substitute samples showed small amounts of FUS-X in one sample (4 µg/kg), as well as FUS-X and ENN B (4 µg/kg and 7 µg/kg, respectively) in another one. A chromatogram showing a spiked gula substitute sample with ENN B is reported in Figure 1. This fact could be attributed to the ingredients used during the gula substitute elaboration process, mainly that from vegetal origin, such as wheat flour, soya, and vegetal protein, among others [24].

Regarding the results obtained in this study, it could be thought that fish processing could be the reason explaining the absence of mycotoxins in smoked fish, as scientific data has shown that food processing or cooking can mitigate mycotoxin contents [25,26]. For instance, in the smoking process, fat is eliminated from fillets, so lipophilic mycotoxins retained in fat portions can be eliminated in this processing step. In further steps, fish fillets are placed in an aqueous solution with a greater amount of salt. Then, it is possible that water-soluble mycotoxins can pass from the fish fillets to the aqueous solution. Salt and sugar are spread over the fillets and placed in a cool place until the salt content reaches the proper level. Finally, the salt is washed with water to stop the curing process [4]. These steps can act as an extraction process. In this way, smoking would naturally eliminate the mycotoxins present in the edible tissues of fish fed with contaminated fish.

Although smoking is the most commonly used fish preservation method, there are different types of smoked fish, depending on the country of consumption. Smoked salmon or trout is commonly consumed in European countries, while in tropical countries, the most consumed one is sun-dried fish. Among different types of smoking, smoke drying method is the most commonly used. One of the main concerns in this type of product is the presence of toxigenic fungi as well as their toxic metabolites, because, as mentioned above, the low moisture product obtained when sun drying is applied presents favorable conditions for fungi growth, which can result in mycotoxin production. In addition, drying under inadequate conditions can also favor the presence of mycotoxins.

As reported in literature, *Aspergillus* species are the predominant species in smoked and dried fish from the tropics [27]. The mycotoxin occurrence in those fish samples could be attributed to: (i) the presence of mycotoxins in fresh fish tissues once the contaminated feed is ingested by farmed fish; and (ii) mycotoxigenic fungal invasionin dried seafood, thus promoting mycotoxin contamination [28].

Compared to mycotoxin determination in terrestrial animals, fewer studies have been performed to determine mycotoxin occurrence in aquatic species. Nevertheless, mycotoxin carry-over from feed to edible fish tissue has been previously reported. In this sense, Huang et al. [21] reported AFB1 contents (2.4–11.8 µg/kg) in muscle and hepatopancreas of gibel carp (*Carassius auratus gibelio*) from subchronic oral administration. In another study, Nomura et al. [22] found AFB1 in edible muscle of rainbow trout. Moreover, they also found higher contents of AFB1 metabolites (aflatoxicol (AFL) and aflatoxin M1 (AFM1)) after dietary exposure in the following raw: AFB1>AFL>AFM1. Despite these results, Nácher-Mestre et al. [6] reported that no fish samples analyzed in their study (gilthead sea bream (*Sparus aurata*) and Atlantic salmon (*Salmo salar*)), were contaminated with mycotoxins after 8 months of feeding with contaminated diets with trichothecenes (mainly deoxynivalenol (DON) from 19.4 µg/kg to 79.2 µg/kg) and FBs (6.4–754.0 µg/kg).

Considering the second option mentioned above related to mycotoxigenic fungal invasion in dried seafood and the consequent mycotoxin production, other previous studies showed the occurrence of mycotoxigenic fungi and also mycotoxins in dried smoked fish. Most of these studies were carried out in tropical and subtropical regions, where high temperature and humidity conditions are essential for fungal growth and proliferation. Temperatures ranging between 25–30 °C and higher humidity were reported to be favorable for fungi growth in smoked-dried fish, with xerophiles being the most commonly associated fungi with low a_w_ dried foods [27].

Moreover, fungi and mycotoxin contamination has been reported in sun-dried fish products. For instance, high levels of ZEN and OTA (317.3 µg/kg and 1.9 µg/kg, respectively) were found in seafood samples. In addition, trace amounts of AFB2 (1.2 µg/kg) were observed in carp muscle after 3 months storage at room temperature [27]. Other authors found that xerophilic molds were the predominant ones in salted fish (molouha) from Egyptian origin [29], with *Aspergillus* spp. being the major one (58.2%), followed by *Penicillium* spp. (32.7%). Moreover, these two fungi genera were also predominant in smoke-cured *Ethmalosa fimbriata* and *Clarias gariepinus* [5].

Likewise, *Aspergillus* species have been reported as the most common fungi associated with smoked fish [30,31,32]. For instance, those fungi were isolated from different fish species and tested for in vitro mycotoxin production, with AFB1, AFB2, AFG1, STG, OTA, and T-2 toxins being the predominant mycotoxins.

Furthermore, other authors also evaluated the AFs concentrations of sun-dried Dagaa fish from Kenya region [33], obtaining AFs concentrations significantly higher than in fresh fish, where no AFs were detected. Therefore, it could be concluded that contamination is produced during drying steps, where toxigenic fungi growth is promoted and incomplete drying favors AFs production. AFs were also detected in smoked-dried fish in Nigeria [34,35,36]. In addition, Job et al. [37] reported strains of *Penicillium digitatum*, *Fusarium equiseti*, and *Fusarium semitectum* as the most predominant in smoke-dried fish sold in Jos Metropolis (Nigeria), at 61.7%, 30.0%, and 26.7% of incidence, respectively.

## 3. Materials and Methods

### 3.1. Chemicals and Reagents

Acetonitrile (MeCN) and methanol (MeOH) were obtained from Merck (Darmstadt, Germany). A Milli-Q water purification system (Millipore Corporation, Bedford, MA, USA) was used to obtain deionized water (<18 MΩ cm resistivity). Ammonium formate (HCO_2_NH_4_, 97%), anhydrous magnesium sulphate, formic acid (HCOOH), mycotoxin stock standard solutions (AFs, OTA, FUS-X, STG, FBs, ENNs, and BEA), and sodium chloride were provided from Sigma-Aldrich (St. Louis, MO, USA). All solvents were filtered through a 0.22 µm cellulose filter (Membrane Solutions, Texas, TX, USA). The working standard solutions consisting of individual compounds were prepared by appropriate dilution of the stock solutions in MeOH or MeCN, depending on its solubility properties, in order to obtain multi-compound working standard solution. The new multianalyte working solution (ranging from 0.1 to 1000 µg/L of concentration for each compound) was stored in darkness conditions in glass-stoppered bottles at −20 °C. The working standard solution consisting in a mixture of the individual compounds was employed for method validation assays.

### 3.2. Procedures

#### 3.2.1. Sampling

To carry out this study, 72 samples of different fish derived products were analyzed: (i) Twenty samples of smoked salmon (*Salmo salar*), (ii) 8 samples of smoked salmon (*Salmo salar*) with dill, (iii) 4 samples of smoked trout (*Onchorhynchus mykiss*), (iv) 30 samples of different types of sushi (*Salmo salar*), and (v) 10 samples of “gula substitute” (fish protein) from different markets located in Valencia (Spain). In Table 3 the ingredients and sample precedence of the samples are shown. The samples were homogenized in a high-speed food blender and analyzed immediately after their acquisition.

#### 3.2.2. Extraction Procedure

The QuEChERS extraction procedure was carried out using a solvent mixture consisting of acidified MeCN/H_2_O. For this purpose, 2 g of a previously homogenized matrix was introduced into a 50-mL polypropylene (PP) centrifugation tube, mixed with 10 mL of water containing 2% of formic acid, and the matrix was allowed to soak for 30 min. Then, 10 mL of MeCN were added into the tube containing the soaked sample and was vigorously shaken on a laboratory shaker (IKA, Staufen, Germany) for 30 min at 250 rpm. In the next step, 4 g of MgSO_4_ and 1 g NaCl were added and shaken immediately to enable uniform distribution of MgSO_4_, and then centrifuged (Eppendorf, Germany) for 5 min (10,000 rpm). Then, 2 mL of MeCN extract were purified by adding 0.1 g of C18 silica sorbent and 0.3 g of MgSO_4_, and centrifuged (10,000 × rpm) for 5 min. The purified extract was filtered and transferred into a vial for further analysis.

#### 3.2.3. Preparation of Standard Solution and Spiking of Blank Samples

Individual stock solutions with a concentration of 1000 µg/kg were prepared in MeCN. They were stored in darkness conditions in glass-stoppered bottles at −20 °C. The working standard solutions consisting of individual compounds were prepared by appropriate dilution of the stock solutions for spiking procedures and calibration curves. Samples of smoked fish and gula substitute containing none of the studied mycotoxins were used as a blank matrix for spiking experiments, as well as for quality control. The spiked samples were left for overnight equilibration.

### 3.3. LC-MS/MS-LIT Analysis

The instrumental analysis was achieved on a LC-MS/MS-LIT system. Chromatographic separation of the analytes was conducted at 25 °C using an Agilent 1200 chromatographic system (Agilent Technologies, Palo Alto, CA, USA) with a binary pump and automatic injector. A reverse-phase Gemini-NX C18 (150 mm×4.6 mm, 5 μm of particle size) analytical column (Phenomenex, Barcelona, Spain) was used. The flow rate was set to 0.2 mL/min, and the oven temperature was 40 °C, with eluent A (water) and eluent B (MeOH) both acidified with 0.1% formic acid and 5 mM ammonium formate. The elution gradient started with 0% of eluent B, increased to 100% in 10 min, decreased to 80% in 5 min, and finally, decreased to 70% in 2 min. During the subsequent 6 min, the column was readjusted to the initial conditions and equilibrated for 7 min. The injection volume was 20 μL.

Regarding mass spectrometry, a 3200 QTRAP^®^ mass spectrometer (AB Sciex, Foster City, CA, USA), which combines a fully functional triple quadrupole and a linear ion trap mass spectrometer within the same instrument, was employed. It was operated in Multiple Reaction Monitoring (MRM) mode and equipped with a turbo electrospray ionization (ESI) interface.A Turbo V^®^ion spray in positive ionization mode (ESI+) was used for the analyses, using nitrogen as the nebulizer and collision gas. The ESI source values were as follows: capillary voltage, 3.50 kV; source temperature, 120 °C; desolvation temperature, 400 °C; cone gas 50 L/h; desolvation gas (nitrogen 99.99% purity) flow, 800 L/h. The resolution for the first and third quadrupoles was set at 12.0 (unit resolution); ion energy, 0.5; entrance and exit energies, 5 and 3, respectively; multiplier, 650; collision gas (argon 99.99% purity) pressure, 3.83 × 10^−3^ mbar; interchanel delay, 0.02 s; total scan time, 1.0 s; dwell time, 0.1 ms. The mass spectrometer was operated in Multiple Reaction Monitoring (MRM) mode in order to obtain the maximum sensitivity for the detection of target molecules. All time measurements were carried out in triplicate. Optimized parameters, such as cone voltages, collision energies, and precursor and product-ions selected, are shown in Table 1.

### 3.4. Method Validation

Method performance parameters were determined according to European guidelines [20]. The method was validated for mycotoxin standards with regards to selectivity, specificity, linearity, matrix effect, sensitivity, trueness, and precision. Representative blank matrices of smoked salmon and gula substitute were selected for validation purposes. Sushi samples were not used for validation purposes as smoked fish was already present as an ingredient in some types of sushi.

The analysis of the standard solutions and the spiked samples allowed determination of the selectivity and specificity of the method. Precursor and product ions and their ratios, as well as RT, were used to confirm the peaks for the targeted compounds in the samples.

Moreover, linearity (expressed as “R^2^”) and matrix effects were studied using standard solutions in neat solvent and matrix-matched calibrations. Different calibration curves (Table 2) for each mycotoxin (0.1/0.5/1/10/20/50/100 µg/L for all the mycotoxins except for FBs (1/10/50/100/250/500/1000 µg/L)) were prepared, to calculate the linearity. In order to establish matrix effects, ratios between the slope of matrix-matched (A) and the slope of external calibration (B) were obtained. Thus, the ratio (A/B) × 100) is defined as matrix effect and expressed as signal suppression/enhancement (SSE, %). SSE values < 100% indicate signal suppression; values > 100% indicate signal enhancement; whereas values equal to 100% indicate no matrix effect. The following formula was used to calculate the signal suppression or enhancement (SSE) due to the matrix effect:

SSE (%) = (slope in matrix/slope in solvent) × 100(1)

Recovery assays were carried out at two different addition levels (20–200 µg/L, for all mycotoxins except for FBs (100–1000 µg/L)). Then, the ratio between the peak area for each mycotoxin from spiked samples before the extraction and those spiked directly in the extract after extraction procedure was calculated. The relative standard deviation (RSD) of the calculated recovery was used to express intraday and interday precision. The RSD for intraday precision was obtained from three determinations for each spiked sample in the same day, while for interday precision, the same approach was used but in 3 different days. LOD and LOQ values were estimated as the lowest-matrix-matched calibration standard corresponding to a signal to noise ratio of at least 3:1 and 10:1, respectively.

### 3.5. Statistics and Data Analysis

All experiments were performed in triplicate, and the results were expressed as the average values ± relative standard deviation (RSD, %).

## 4. Conclusions

From the results obtained in the present study, it can be concluded that a LC-MS/MS-LIT method was optimized and validated for a simultaneous multi-mycotoxin determination in ready-to-eat processed samples of fish, including smoked fish, sushi, and gula substitute. Mycotoxins were not found in smoked salmon, trout, or sushi samples. However, ENN B and FUS-X were detected in gula substitute, which can be explained by the use of contaminated products or ingredients in their elaboration or due to improper elaboration or storage conditions. Thus, adequate elaboration procedures and storage conditions, mainly cold chain maintenance, are essential to preserve fish food products from fungal and mycotoxin contamination.

## Figures and Tables

**Table 1:** Optimized parameters for the studied mycotoxins.

MYCOTOXIN	DP ^a^	PRECURSOR ION *m*/*z*	PRODUCT ION ^Q^			PRODUCT ION ^q^		
			CE ^b^	ION	CXP ^c^	CE ^b^	ION	CXP ^c^
**AFB1**	46	313.1 [M + H]^+^	41	241.0	4	39	289.9	4
**AFB2**	81	315.1 [M + H]^+^	33	286.9	6	39	259.0	6
**AFG1**	76	329.2 [M + H]^+^	39	243.1	6	29	311.1	6
**AFG2**	61	331.1 [M + H]^+^	27	313.1	6	39	245.1	4
**FB1**	101	722.2 [M + H]^+^	51	334.2	20	45	352.2	26
**FB2**	131	706.2 [M + H]^+^	50	336.3	16	50	318.3	18
**FB3**	100	706.5 [M + H]^+^	50	336.3	16	50	318.4	18
**FUS-X**	47	355 [M + H]^+^	45	175.0	3	45	246.7	3
**ENN A**	76	699.4 [M + NH_4_]^+^	59	228.2	16	35	210.1	14
**ENN A1**	66	685.4 [M + NH_4_]^+^	59	214.2	10	37	210.2	8
**ENN B**	51	657.3 [M + NH_4_]^+^	39	196.1	8	59	214.0	10
**ENN B1**	66	671.2 [M + NH_4_]^+^	61	214.2	10	57	228.1	12
**BEA**	116	801.2 [M + NH_4_]^+^	27	784.1	10	39	244.1	6
**OTA**	55	404.3 [M + H]^+^	97	102.1	6	27	239.0	6
**STG**	106	325 [M + H]^+^	51	281.0	18	50	310.0	3

Note: *m*/*z* = mass/charge; ^a^ DP = Declustering Potential; ^Q^ = Quantitation transition; ^q^ = Qualification transition; ^b^ = Collision Energy (CE); ^c^ = Collision Cell Exit Potential (CXP). All expressed in voltage (V).

**Table 2:** Retention time (RT), recovery, limits of detection (LOD) and quantitation (LOQ), matrix effect (expressed as SSE), and calibration curves for each analyzed mycotoxin.

Mycotoxin	RT (min)	Recovery (%) *	LOD (µg/kg) *	LOQ (µg/kg) *	SSE *	Calibration Curve
AFB1	9.35	92.1	1.0	3.0	93.9	y = 351,486x + 26,4429
AFB2	9.23	84.0	1.0	3.0	93.5	y = 2 × 10^6^x + 17,6671
AFG1	9.07	111.0	2.0	7.0	92.9	y = 2 × 10^6^x + 16,5804
AFG2	8.95	108.5	2.0	7.0	89.3	y = 1 × 10^6^x + 10,7126
FB1	9.33	80.4	10.0	33.3	56.3	y = 69,965x − 885.42
FB2	9.85	97.4	10.0	33.3	58.7	y = 75,234x − 429.53
FB3	9.64	107.0	10.0	33.3	76.5	y = 59,893x + 651.29
FUS-X	13.17	125.0	1.0	4.0	99.8	y = 36,631x − 8066.7
ENN A	18.35	107.7	1.0	5.0	82.8	y = 2 × 10^6^x + 29,6486
ENN A1	18.10	119.2	1.0	4.0	88.1	y = 9 × 10^6^x + 62,446
ENN B	17.00	116.6	1.0	4.0	84.7	y = 2 × 10^7^x + 62,836
ENN B1	17.34	97.7	1.0	4.0	83.2	y = 1 × 10^6^x + 133,029
BEA	17.65	109.5	5.0	10.0	81.7	y = 24,026x + 4862.8
OTA	10.53	85.0	1.0	4.0	96.7	y = 532,784x + 17,528
STG	10.90	90.4	10.0	33.3	72.4	y = 192,544x − 1991.7

Note: * Analyses performed in triplicate.

**Table 3:** Samples included in the study.

Sample	Presentation (Number of Samples)	Type/Origin	Ingredients
Atlantic Salmon (*Salmo salar*)	Smoked salmon (*n* = 20)	Smoked/Norway	Atlantic salmon (97%), water, salt, sugar, natural smoke, antioxidant E-331, E-501, E-262
	Smoked salmon with dill (*n* = 8)	Smoked/Norway	Atlantic salmon (97%), salt, natural smoke, brandy, dill
Rainbow Trout (*Onchorhynchus mikyss*)	Smoked trout (*n* = 4)	Smoked/Spain, France	Trout, salt, sugar, natural smoke
Sushi Atlantic Salmon (*Salmo salar*)	Sushi Nigiri (*n* = 10)	Crude/Norway	Rice (rice, water, sugar, sunflower oil, cane molasses, trehalose), salmon
	Sushi Maki (*n* = 10)	Crude/Norway	Salmon, rice (water, rice vinegar, sugar, salt), nori algae, cucumber
	Sushi California Roll (*n* = 5)	Crude/Norway	Rice (water, rice vinegar, sugar, salt), nori algae, cucumber, cheese, onion
	Salmon for sushi (*n* = 5)	Crude/Norway	Salmon (*Salmo salar*), salt, natural flavor, sugar
Gula substitute	(*n* = 10)	Crude/Spain	Surimi 47% (fish, cephalopods), water, sunflower oil, corn starch, modified starches (gluten free), aroma (soybean, traces of crustaceans) and fish extract, egg white, vegetable protein gluten), salt, flavor enhancer (monosodium glutamate, E-635), stabilizer (xanthan gum), sepia (mollusk) ink.
